# Comparison of lower extremity atherosclerosis in diabetic and non-diabetic patients using multidetector computed tomography

**DOI:** 10.1186/1471-2261-14-125

**Published:** 2014-09-24

**Authors:** Ci He, Jin-gang Yang, Yun-ming Li, Jian Rong, Fei-zhou Du, Zhi-gang Yang, Ming Gu

**Affiliations:** Department of Radiology, Chengdu Military General Hospital, Chengdu, Sichuan 610083 China; Department of Neurosurgery, Chengdu Military General Hospital, Chengdu, Sichuan 610083 China; Division of Geriatric Medicine, Department of Medicine, Chengdu Military General Hospital, Chengdu, Sichuan 610083 China; Department of Radiology, West China Hospital, Sichuan University, Chengdu, Sichuan 610041 China

**Keywords:** Diabetes mellitus, Lower extremity, Atherosclerosis, Computed tomography, Angiography

## Abstract

**Background:**

Lower extremity atherosclerosis (LEA) is among the most serious diabetic complications and leads to non-traumatic amputations. The recently developed dual-source CT (DSCT) and 320- multidetector computed tomography (MDCT) may help to detect plaques more precisely. The aim of our study was to evaluate the differences in LEA between diabetic and non-diabetic patients using MDCT angiography.

**Methods:**

DSCT and 320-MDCT angiographies of the lower extremities were performed in 161 patients (60 diabetic and 101 non-diabetic). The plaque type, distribution, shape and obstructive natures were compared.

**Results:**

Compared with non-diabetic patients, diabetic patients had higher peripheral neuropathy, history of cerebrovasuclar infarction and hypertension rates. A total of 2898 vascular segments were included in the analysis. Plaque and stenosis were detected in 681 segments in 60 diabetic patients (63.1%) and 854 segments in 101 non-diabetic patients (46.9%; *p* <0.05). Regarding these plaques, diabetic patients had a higher incidence of mixed plaques (34.2% vs. 27.1% for non-diabetic patients). An increased moderate stenosis rate and decreased occlusion rate were observed in diabetic patients relative to non-diabetic patients (35.8% vs. 28.3%; and 6.6% vs. 11.4%; respectively). In diabetic patients, 362 (53.2%) plaques were detected in the distal lower leg segments, whereas in non-diabetic patients, 551 (64.5%) plaques were found in the proximal upper leg segments. The type IV plaque shape, in which the full lumen was involved, was detected more frequently in diabetic patients than in non-diabetic patients (13.1% vs. 8.2%).

**Conclusion:**

Diabetes is associated with a higher incidence of plaque, increased incidence of mixed plaques, moderate stenosis and localisation primarily in the distal lower leg segments. The advanced and non-invasive MDCT could be used for routine preoperative evaluations of LEA.

## Background

According to statistics from the International Diabetes Federation [1], the global diabetes prevalence reached 246 million in 2007, more than double the rate from the previous decade. It has been predicted that by 2025, the global diabetic patient population will reach 380 million [2]. Lower extremity atherosclerosis (LEA) is among the most serious diabetic complications and leads to non-traumatic amputations [3]. The risk of amputation is 15–46-fold higher among diabetic patients than among non-diabetic patients [4]. Meanwhile, diabetes is complicated by cerebral vascular disease and coronary heart disease (CHD); therefore, the mortality of diabetic patients is significantly increased [5].

LEA is always insidious, and it is very important to conduct early and accurate imaging evaluations in diabetic patients to improve patient outcomes, reduce the amputation rates and amputation planes and reduce the treatment costs [6]. Digital subtraction angiography (DSA) is currently considered the ‘gold standard’ for vascular disease diagnosis, and can offer interventional treatments at the same time; however, this technique is invasive, expensive, requires highly trained surgical staff and is potentially dangerous [7]. Regarding the development of imaging technologies, multidetector computed tomography (MDCT) has been widely used for non-invasive vascular imaging evaluations [8–10]. The recently introduced dual-source CT (DSCT) and 320-MDCT has been widely used to evaluate cardiovascular and head and neck vascular lesions [11–13]; however, few reports have described their usefulness for LEA lesions. The purposes of this study were to explore the application of MDCT angiography for LEA and evaluate the differences in LEA plaque prevalence and morphology between diabetic and non-diabetic patients.

## Methods

### Study patients

From November 2011 to November 2013, we retrospectively observed a total of 60 consecutive diabetic patients (13 women; mean age, 69.42 ± 11.04 years) and 101 non-diabetic patients (23 women; mean age, 68.50 ± 13.59 years) who underwent DSCT and 320-MDCT angiography of the arteries in both legs. The exclusion criteria included an allergy to the iodine contrast agent, liver, kidney or heart failure (Creatinine level ≥120 mol/L), pregnancy and leg amputation. The vascular exclusion criteria included vascular malformations, poor imaging and a lumen diameter <1.5 mm.

Baseline demographics and medical history were provided, such as age, gender, history of diabetes mellitus, hypertension, CHD, cerebrovasuclar infarction (CI) and laboratory tests. All subjects provided informed consent, and the study was approved by the ethics committees of West China Hospital and Military General Hospital of Chengdu PLA.

### MDCT scanning

Examinations were performed with DSCT (Somatom Definition; Siemens Medical Solutions, Forchheim, Germany) (n = 136, from November 2011 to June 2013) at West China Hospital, and 320-MDCT (Aquilion one, Toshiba Medical Systems, Tokyo, Japan) (n = 25, from May 2013 to November 2013) at Military General Hospital of Chengdu PLA. The scan parameters of DSCT were as follows: tube voltages, 120 KV and 80 KV; tube currents, 55 mAs and 230 mAs; collimation, 2 × 64 × 0.6 mm; pitch, 0.65; reconstruction thickness, 0.75 mm and interlayer spacing, 0.4 mm. CT data were acquired in the craniocaudal direction from the common iliac artery to the plantar plane. The delay between contrast injection and CT acquisition was determined using bolus tracking software. A circular region of interest (ROI) for attenuation measurement was placed in the common iliac artery; data acquisition was initiated as soon as the signal intensity in this ROI reached a threshold of 100 Hounsfield units (HU). A non-ionic contrast medium (80–100 mL of iopamidol, 370 mg iodine/mL; Bracco Sine Pharmaceutical Corp. Ltd., Shanghai, China) was immediately administered, followed by 40 mL of a saline chaser solution through an 18-gauge intravenous antecubital catheter with a dual-head power injector (Stellant; Medrad, Indianola, PA, USA) at a flow rate of 6 mL/s. 320-MDCT examination was obtained following standard protocols similar to DSCT.

### Image reconstruction

The images were simultaneously transferred to 3D post-processing workstation 1 (Syngo-Imaging; Siemens Medical Solutions, Forchheim, Germany) and workstation 2 (Aquilion one, Toshiba Medical Systems, Tokyo, Japan). Post-processing reconstruction was performed on the workstation and incorporated multi-planar reconstruction (MPR), maximum intensity projection (MIP), volume rendering (VR) and curved planar reformation (CPR).

### Image analysis

**Figure 1:**
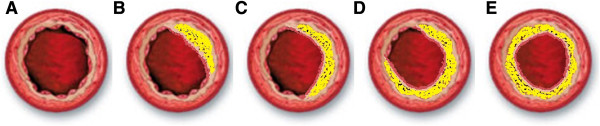
**Plaque shapes. A**. Normal lumen. **B**. Type I, <25%; **C**. Type II, 25–50%; **D**. Type III, 50–75%; **E**. Type IV, 75–100%.

The bilateral lower extremity arteries were divided into 18 segments, or 9 per leg; these were the common iliac artery, internal iliac artery, external iliac artery, femoral artery, popliteal artery, anterior tibial artery, posterior tibial artery, peroneal artery and dorsalis pedis artery. These segments were also divided into 2 categories to include the upper leg arteries (common iliac artery, internal iliac artery, external iliac artery and femoral artery) and lower leg arteries (popliteal artery, anterior tibial artery, posterior tibial artery, peroneal artery and dorsalis pedis artery). The plaques were classified as non-calcified (<50 HU), mixed (60–100 HU) or calcified (>130 HU) according to the average HU value [14]. Luminal narrowing values were automatically calculated by the software. The artery stenosis grade was classified as mild stenosis (luminal narrowing, <50%), moderate stenosis (luminal narrowing, 50%–74%), severe stenosis (luminal narrowing, ≥75%) and occlusion (luminal narrowing, 100%) [15]. The atherosclerosis artery axial plane was divided into 4 quadrants, and the plaque shapes were described as type I, <25%; type II, 25–50%; type III, 50–75% and type IV, 75–100% (Figure 1).

Two experienced radiologists blinded to the diagnostic indices and each other’s decisions evaluated the reconstructed images for plaque distribution and properties. Only in cases of disagreement did the 2 radiologists discuss a case to reach a decision.

### Statistical methods

The clinical information, clinical symptoms, laboratory tests, number of diseased segments, types and shapes of plaques and grades of luminal narrowing were analysed statistically in each patient. Kolmogorov–Smirnov test was used to test the normality of the distribution. Continuous data are given as means ± standard deviations. Continuous variables such as laboratory tests were expressed as means ± standard deviations and were compared using Student’s t-test for unpaired data or the Mann–Whitney two-sample statistic,as appropriate. Categorical variables such as the types and shapes of plaques were presented as numbers (percentages) and were compared using the χ^2^ test. All data were analysed using the SPSS 16.0 statistical software package for Windows XP (SPSS Inc., Chicago, IL, USA). A p-value of <0.05 was considered statistically significant.

## Results

### General information

All examinations were successfully completed in all patients without the occurrence of any complications, and all examinations were of diagnostic image quality with good to excellent vessel visibility.

Compared with non-diabetic patients, diabetic patients had higher peripheral neuropathy, hypertension and history of CI incidence rates (*p* < 0.05). The remaining clinical data and laboratory test results are shown in Table 1.

**Table 1:** **Characteristics of study population**

Characteristic	Non diabetic (n = 101)	Diabetic (n = 60)	***P***
Age (years)	68.50 ± 13.59	69.42 ± 11.04	>0.05
Gender (female)	23 (22.8%)	13 (21.7%)	>0.05
Smoking	43 (42.6%)	26 (43.3%)	>0.05
BMI	21.28 ± 2.9	22.34 ± 4.11	>0.05
Blood glucose (mmol/L)	5.92 ± 1.54	7.98 ± 1.42	0.001
Cholesterol (mmol/L)	3.94 ± 1.09	4.39 ± 1.18	0.017
Triglyceride (mmol/L)	1.33 ± 0.74	1.57 ± 0.74	0.042
HDL-C (mmol/L)	1.14 ± 0.31	1.12 ± 0.24	>0.05
LDL-C (mmol/L)	2.27 ± 0.86	2.52 ± 0.90	>0.05
Creatinine (mmol/L)	83.04 ± 25.53	82.39 ± 31.19	>0.05
Uric acid	311.57 ± 100.07	331.48 ± 105.26	>0.05
Hypertension	53 (52.5%)	42 (70.0%)	0.029
Peripheral neuropathy	22 (21.8%)	25 (41.7%)	0.007
History of CHD	29 (28.7%)	22 (36.7%)	>0.05
History of CI	72 (33.7%)	36 (60.0%)	0.001

Note: Data were expressed as n (%) or mean ± S.D.

*BMI*: body mass index; *HDL*-*C*: high density lipoprotein cholesterol; *LDL*-*C*: low density lipoprotein cholesterol. *CHD*: coronary heart disease; *CI*: cerebrovasuclar infarction.

### Plaque type and stenosis degree

**Figure 2:**
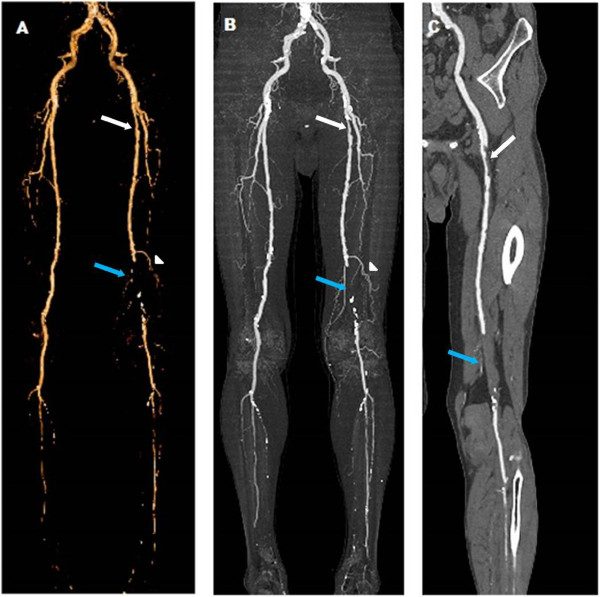
**DSCT images of a 56** **-year-** **old man with diabetes for 15 years** **, 1 year of cold feet,** **show diffuse plaques and stenoses in both lower extremities. A**, Volume-rendered reconstruction (VRT) after dual energy bone removal displays overview of both lower extremities with a mild stenosis (white arrow), an occlusion (blue arrow) and a compensatory artery (white triangle) in the right femoral artery. **B**, Maximum intensity projection (MIP) depicts the overview of plaques and stenosis. **C**, Both the mild stenosis (white arrow) and occlusion (blue arrow) are caused by non-calcified plaque as evidenced using curved planar reformation (CPR).

A total of 2898 vascular segments were included in the analysis. Plaque and stenosis were detected in 681 (63.1%) vessel segments in 60 diabetic patients and 854 (46.9%) vessel segments in 101 non-diabetic patients (*p* < 0.001). There was a statistical difference in plaque type between diabetic and non-diabetic patients (*p* < 0.05), and diabetic patients had a higher incidence of mixed plaques (Figure 2, Table 2).

**Table 2:** **Comparison of plaque and stenosis between diabetic and non**-**diabetic patients**

Characteristics	Non-diabetic	Diabetic	***P***
N	854 (46.9%)	681 (63.1%)	<0.001
**Plaque type**			0.007
Non-calcified	217 (25.4%)	144 (21.2%)	
Mixed	231 (27.1%)	233 (34.2%)	
Calcified	406 (47.5%)	304 (44.6%)	
**Grade of stenosis**			<0.001
Mild (<50%)	363 (42.5%)	299 (43.9%)	
Moderate (≥50%)	242 (28.3%)	244 (35.8%)	
Severe (≥75%)	152 (17.8%)	93 (13.7%)	
Occlusion (=100%)	97 (11.4%)	45 (6.6%)	
**Plaque shape**			0.018
Type I < 25%	130 (15.2%)	91 (13.4%)	
Type II < 50%	377 (44.2%)	290 (42.6%)	
Type III < 75%	277 (32.4%)	211 (30.9%)	
Type IV ≤ 100%	70 (8.2%)	89 (13.1%)	

Note: Data were expressed as n (%).

**Figure 3:**
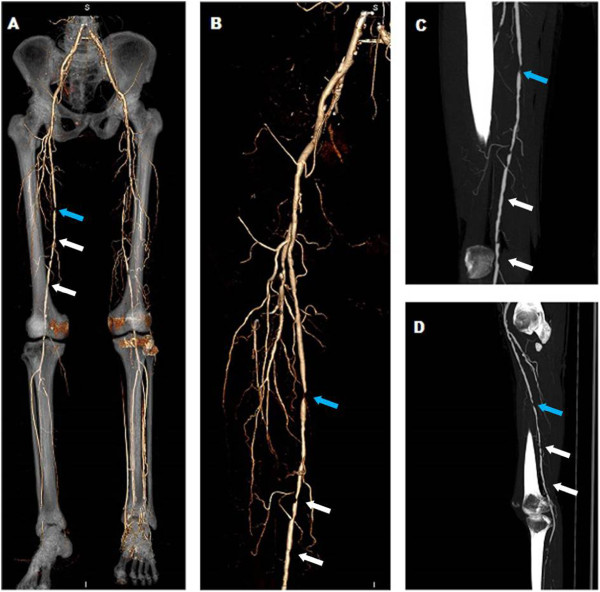
**320**-**MDCT images of a 62** **-year-** **old man with diabetes for 10 years** **, intermittent claudication of both lower extremities for 6 months. A**, VRT reflects overview artery tree of lower extremities with bone remaining, showing diffused stenoses in right femoral artery. **B**, VRT image after bone removal depicts a severe (blue arrow) and diffused mild to moderate stenosis (white arrows) in right femoral artery. **C**, All the stenoses are caused by non-calcified plaques as evidenced using coronal MIP. **D**, Sagital MIP displays the severe stenosis (blue arrow), mild to moderate stenoses (white arrows) in right femoral artery caused by non-calcified plaques.

Statistical difference was observed in the degree of stenosis between the 2 groups (*p* < 0.05). Compared with non-diabetic patients, diabetic patients had a higher incidence of moderate stenosis (35.8% vs. 28.3%) and a lower incidence of occlusion (6.6% vs. 11.4%), as shown in Table 2, Figure 3.

### Plaque distribution and shape

**Figure 4:**
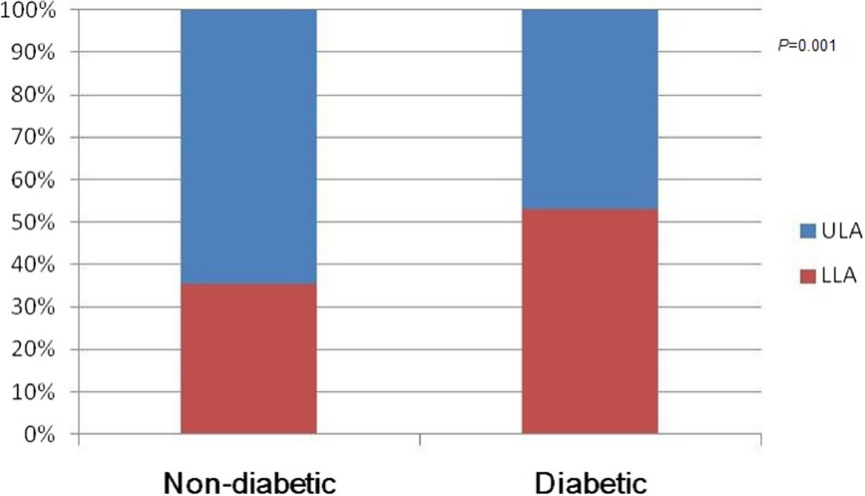
**Bar graph demonstrates the main distribution of plaques between diabetic and non**-**diabetic patients**, **showing the increased distal segment involvement in diabetic patients (** ***P =*** **0.01)** **.** ULA = Upper Leg arteries, LLA = Lower leg arteries.

Regarding plaque distribution, 362 (53.2%) plaques were detected in the distal lower leg segments of diabetic patients, and there was an increased involvement in the distal lower leg segments, particularly in the popliteal artery, anterior tibial artery and posterior tibial artery. In non-diabetic patients, 551 (64.5%) plaques were found in the proximal upper leg segments. The increased distal segment involvement in diabetic patients and increased proximal segment involvement in non-diabetic patients represented significant differences (*p* = 0.001; Figure 4).

Extensive plaques were observed in both diabetic and non-diabetic patients; the plaque shapes were primarily classified as type II and type III, although the incidence of type IV, in which the full lumen is involved, was higher among diabetic patients than among non-diabetic patients and this difference was statistically significant (Table 2).

## Discussion

LEA is a common complication of diabetes [16, 17]. Dormandy et al. [16] reported that the prevalence of LEA among type 2 diabetic patients was as high as 23.5%. In our study, there were no significant differences with respect to age, sex, smoking status, and uric acid between diabetic and non-diabetic patients. Blood glucose levels, history of CI and peripheral neuropathy and hypertension incidence rates were significantly higher among diabetic patients, indicating that blood sugar and blood pressure statuses correlated closely with atherosclerosis development. These statuses also explained the more severe atherosclerotic lesions observed among diabetic patients relative to non-diabetic patients, a finding that corroborated relevant reports [18–20].

Our study found that the incidence of LEA was significantly higher among diabetic patients than among non-diabetic patients. In our study, a higher incidence of mixed plaques was observed in the diabetic group, a finding that was consistent with previous MDCT-based studies [21, 22]. Rosamond et al. [22] reported that type 2 diabetes often led to the development of multiple atherosclerotic plaques, especially unstable mixed plaques. Unstable mixed plaques, which feature reduced calcification and increased fibrotic and lipid contents, are more vulnerable and more easily form ulcers and ruptures that lead to thrombosis, acute coronary heart syndrome and other serious and possibly life-threatening complications [23].

Artery stenoses were mainly mild to moderate in both groups. Compared with non-diabetic patients, diabetic patients had a higher incidence of moderate stenosis and a lower incidence of occlusion. Diabetes-induced atherosclerosis was primarily non-obstructive stenosis. Scholte et al. [2] evaluated the diabetes-related coronary atherosclerotic plaque and morphological statuses using 64-slice CT and found that approximately 82% of stenoses in diabetic patients were non-occlusive lesions, in accordance with our study. Other reports of ultrasound and CT studies [10, 21] indicated that when compared with those in non-diabetic patients, atherosclerotic plaques in diabetic patients, which were mainly mixed plaques with reduced occlusion, were more unstable and thus more easily ruptured and thrombosed.

Our study found that the 2 groups differed significantly in terms of plaque distribution. Non-diabetic atherosclerosis primarily affected the proximal upper leg segments. In contrast, an increased involvement in the distal lower leg segments was observed in diabetic patients, particularly in the popliteal artery, anterior tibial artery and posterior tibial artery. Previous studies [24, 25] have supported the finding that diabetes-induced atherosclerosis involved the distal segments; in particular, the distal segment atherosclerosis rate was as high as 58% among diabetic patients and was accompanied by more severe stenosis and an increased tendency toward vascular embolism. It was unclear why atherosclerosis distributions differed between diabetic and non-diabetic patients. We speculate that diabetes might alter the arterial remodelling process, thus resulting in diffusely narrowed arteries that lack compensatory enlargement, especially in distal segments.

In our study, we also found that plaque shapes in both groups were primarily type II and type III. The incidence of Type IV was higher among diabetic patients than among non-diabetic patients, indicating more widespread atherosclerosis among diabetic patients. Diabetic-induced atherosclerosis was usually multi-stage and widespread and beaded changes in the distal segments [16, 18]. These characteristics might induce relatively more severe symptoms, a poorer prognosis and a higher amputation incidence. The more diffuse atherosclerotic burdens in diabetic patients were likely caused by the increased risk factors resulting from the metabolic disorders that have been described to cause atherosclerosis [26, 27].

When considering non-invasive imaging, the magnetic resonance (MR) imaging and ultrasound results should also be compared. Following the developments in non-invasive imaging equipment and technology, ultrasound and MR are increasingly used in examinations of lower extremity arterial disease [28]. However, ultrasound analysis is operator-dependent. MR imaging, which features various imaging protocols and sequences, has a demonstrated ability to characterise morphological, structural and compositional features of atherosclerotic plaques in vivo [29]. However, this method is time-consuming and cannot be performed on some patients with pacemakers or stents. MDCT has undergone rapid development. In particular, dual-source CT and 320-MDCT have many advantages relative to conventional MDCT, such as higher temporal and spatial resolution, radiation dose reduction [30], and powerful post-processing capabilities.

This study has the following shortcomings. CT angiography has some limitations with respect to accurate determination of stenoses involving large plaques in distal small segments. Although the radiation dose provided by DSCT and 320-MDCT is significantly reduced, multiple inspections cannot be performed within a short time period.

## Conclusion

The incidence of LEA was higher among diabetic patients than non-diabetic patients. Diabetic atherosclerosis, which featured a higher incidence of mixed plaque and moderate stenosis, was more extensive in the distal segments and primarily involved arteries in the lower leg. As an advanced non-invasive technique, MDCT could provide good evaluations of LEA plaque types, shapes, distributions and stenosis characteristics and could therefore be used for routine preoperative evaluations.

## References

[1] Unwin N, Gan D, Whiting D (2010). The IDF Diabetes Atlas: providing evidence, raising awareness and promoting action. Diabetes Res Clin Pract.

[2] Scholte AJ, Schuijf JD, Kharagjitsingh AV, Jukema JW, Pundziute G, van der Wall EE, Bax JJ (2008). Prevalence of coronary artery disease and plaque morphology assessed by multi-slice computed tomography coronary angiography and calcium scoring in asymptomatic patients with type 2 diabetes. Heart.

[3] Ohnishi H, Sawayama Y, Furusyo N, Maeda S, Tokunaga S, Hayashi J (2010). Risk factors for and the prevalence of peripheral arterial disease and its relationship to carotid atherosclerosis: the Kyushu and Okinawa Population Study (KOPS). J Atheroscler Thromb.

[4] Nguyen LL, Hevelone N, Rogers SO, Bandyk DF, Clowes AW, Moneta GL, Lipsitz S, Conte MS (2009). Disparity in outcomes of surgical revascularization for limb salvage: race and gender are synergistic determinants of vein graft failure and limb loss. Circulation.

[5] Roper NA, Bilous RW, Kelly WF, Unwin NC, Connolly VM (2001). Excess mortality in a population with diabetes and the impact of material deprivation: longitudinal, population based study. BMJ.

[6] Harrington C, Zagari MJ, Corea J, Klitenic J (2000). A cost analysis of diabetic lower-extremity ulcers. Diabetes Care.

[7] Willinsky RA, Taylor SM, TerBrugge K, Farb RI, Tomlinson G, Montanera W (2003). Neurologic complications of cerebral angiography: prospective analysis of 2,899 procedures and review of the literature. Radiology.

[8] Kock MC, Dijkshoorn ML, Pattynama PM, Myriam Hunink MG (2007). Multi-detector row computed tomography angiography of peripheral arterial disease. Eur Radiol.

[9] Mekle R, Hofmann E, Scheffler K, Bilecen D (2006). A polymer-based MR-compatible guidewire: a study to explore new prospects for interventional peripheral magnetic resonance angiography (ipMRA). J Magn Reson Imaging.

[10] Verim S, Tasci I (2013). Doppler ultrasonography in lower extremity peripheral arterial disease. Turk Kardiyol Dern Ars.

[11] Pflederer T, Marwan M, Renz A, Bachmann S, Ropers D, Kuettner A, Anders K, Bamberg F, Daniel WG, Achenbach S (2009). Noninvasive assessment of coronary in-stent restenosis by dual-source computed tomography. Am J Cardiol.

[12] He C, Yang ZG, Chu ZG, Dong ZH, Shao H, Deng W, Chen J, Peng LQ, Tang SS, Xiao JH (2010). Carotid and cerebrovascular disease in symptomatic patients with type 2 diabetes: assessment of prevalence and plaque morphology by dual-source computed tomography angiography. Cardiovasc Diabetol.

[13] He C, Yang ZG, Chu ZG, Dong ZH, Li YM, Shao H, Deng W (2010). Comparison of carotid and cerebrovascular disease between diabetic and non-diabetic patients using dual-source CT. Eur J Radiol.

[14] Ballotta E, Da Giau G, Renon L (2000). Carotid plaque gross morphology and clinical presentation: a prospective study of 457 carotid artery specimens. J Surg Res.

[15] North American Symptomatic Carotid Endarterectomy Trial Collaborators (1991). Beneficial effect of carotid endarterectomy in symptomatic patients with high-grade carotid stenosis. N Engl J Med.

[16] Dormandy JA, Betteridge DJ, Schernthaner G, Pirags V, Norgren L (2009). Impact of peripheral arterial disease in patients with diabetes–results from PROactive (PROactive 11). Atherosclerosis.

[17] Pomposelli F (2010). Arterial imaging in patients with lower-extremity ischemia and diabetes mellitus. J Am Podiatr Med Assoc.

[18] Federman DG, Kravetz JD (2007). Peripheral arterial disease: diagnosis, treatment, and systemic implications. Clin Dermatol.

[19] Cardoso CR, Leite NC, Freitas L, Dias SB, Muxfeld ES, Salles GF (2008). Pattern of 24-hour ambulatory blood pressure monitoring in type 2 diabetic patients with cardiovascular dysautonomy. Hypertens Res.

[20] Cacoub PP, Abola MT, Baumgartner I, Bhatt DL, Creager MA, Liau CS, Goto S, Rother J, Steg PG, Hirsch AT (2009). Cardiovascular risk factor control and outcomes in peripheral artery disease patients in the Reduction of Atherothrombosis for Continued Health (REACH) Registry. Atherosclerosis.

[21] Ibebuogu UN, Nasir K, Gopal A, Ahmadi N, Mao SS, Young E, Honoris L, Nuguri VK, Lee RS, Usman N, Rostami B, Pal R, Flores F, Budoff MJ (2009). Comparison of atherosclerotic plaque burden and composition between diabetic and non diabetic patients by non invasive CT angiography. Int J Cardiovasc Imaging.

[22] Rosamond W, Flegal K, Furie K, Go A, Greenlund K, Haase N, Hailpern SM, Ho M, Howard V, Kissela B, Kittner S, Lloyd-Jones D, McDermott M, Meigs J, Moy C, Nichol G, O'Donnell C, Roger V, Sorlie P, Steinberger J, Thom T, Wilson M, Hong Y (2008). Heart disease and stroke statistics–2008 update: a report from the American Heart Association Statistics Committee and Stroke Statistics Subcommittee. Circulation.

[23] Naghavi M, Libby P, Falk E, Casscells SW, Litovsky S, Rumberger J, Badimon JJ, Stefanadis C, Moreno P, Pasterkamp G, Fayad Z, Stone PH, Waxman S, Raggi P, Madjid M, Zarrabi A, Burke A, Yuan C, Fitzgerald PJ, Siscovick DS, de Korte CL, Aikawa M, Airaksinen KE, Assmann G, Becker CR, Chesebro JH, Farb A, Galis ZS, Jackson C, Jang IK (2003). From vulnerable plaque to vulnerable patient: a call for new definitions and risk assessment strategies: Part II. Circulation.

[24] Carter A, Murphy MO, Turner NJ, Halka AT, Ghosh J, Serracino-Inglott F, Walker MG, Syed F (2007). Intimal neovascularisation is a prominent feature of atherosclerotic plaques in diabetic patients with critical limb ischaemia. Eur J Vasc Endovasc Surg.

[25] van der Feen C, Neijens FS, Kanters SD, Mali WP, Stolk RP, Banga JD (2002). Angiographic distribution of lower extremity atherosclerosis in patients with and without diabetes. Diabet Med.

[26] Wang TD, Goto S, Bhatt DL, Steg PG, Chan JC, Richard AJ, Liau CS (2010). Ethnic differences in the relationships of anthropometric measures to metabolic risk factors in Asian patients at risk of atherothrombosis: results from the REduction of Atherothrombosis for Continued Health (REACH) Registry. Metabolism.

[27] Escobedo J, Schargrodsky H, Champagne B, Silva H, Boissonnet CP, Vinueza R, Torres M, Hernandez R, Wilson E (2009). Prevalence of the metabolic syndrome in Latin America and its association with sub-clinical carotid atherosclerosis: the CARMELA cross sectional study. Cardiovasc Diabetol.

[28] Kreitner KF, Schmitt R (2007). MultiHance-enhanced MR angiography of the peripheral run-off vessels in patients with diabetes. Eur Radiol.

[29] Lell M, Fellner C, Baum U, Hothorn T, Steiner R, Lang W, Bautz W, Fellner FA (2007). Evaluation of carotid artery stenosis with multisection CT and MR imaging: influence of imaging modality and postprocessing. AJNR Am J Neuroradiol.

[30] Zhang LJ, Wu SY, Niu JB, Zhang ZL, Wang HZ, Zhao YE, Chai X, Zhou CS, Lu GM (2010). Dual-energy CT angiography in the evaluation of intracranial aneurysms: image quality, radiation dose, and comparison with 3D rotational digital subtraction angiography. AJR Am J Roentgenol.

[CR31] The pre-publication history for this paper can be accessed here:http://www.biomedcentral.com/1471-2261/14/125/prepub

